# Overexpression of TROP2 Predicts Poor Prognosis of Patients with Cervical Cancer and Promotes the Proliferation and Invasion of Cervical Cancer Cells by Regulating ERK Signaling Pathway

**DOI:** 10.1371/journal.pone.0075864

**Published:** 2013-09-27

**Authors:** Ting Liu, Yueyang Liu, Xiangxiang Bao, Jiguang Tian, Yang Liu, Xingsheng Yang

**Affiliations:** 1 Department of Obstetrics and Gynecology, Qilu Hospital of Shandong University, Jinan, Shandong, China; 2 Department of Orthopedics, Qilu Hospital of Shandong University, Jinan, Shandong, China; Florida International University, United States of America

## Abstract

Overwhelming evidence has demonstrated that the aberrant expression of the human trophoblast cell-surface antigen (TROP2) was associated with tumor aggressiveness and poor prognosis in a variety of human cancers, however the roles of TROP2 in cervical cancer have not been investigated. The purpose of our study was to elucidate the prognostic significance of TROP2 expression in patients with cervical cancer and determine its effect on tumor progression. Immunohistochemistry assay showed that 88.7% (94/106 cases) of cervical cancer specimens were positively stained with TROP2, and the overexpression of TROP2 was closely related with FIGO stage, histological grades, lymphatic metastasis, invasive interstitial depth and high expression of Ki-67. Patients with TROP2-positive staining exhibited a significantly decreased overall survival and progression free survival; it was also an independent predictor for prognosis according to multivariate analysis. Moreover, down-regulation of TROP2 mediated by siRNA in Siha and CaSki cells resulted in a strong inhibition of proliferation and invasion, TROP2 abrogation also elevated the apoptotic ratio and caused G1 arrest. Conversely, enforced expression of TROP2 in HeLa and C33A cells remarkably promoted cell growth, migration and invasion. In addition, the tumorigenic function of TROP2 was associated with the increased expressions of cyclin D1, cyclin E, CDK2 and CDK4 but reduced expression of p27 and E-cadherin via the activation of Erk1/2 signaling pathway. Furthermore, the inhibition of TROP2 expression in cervical cancer cell lines enhances sensitivity to cisplatin. The present study suggest that overexpression of TROP2 may play crucial roles in the development and pathogenesis of human cervical cancer, therefore, TROP2 may represent a prospective prognostic indicator and a potential therapeutic target of cervical cancer.

## Introduction

Cervical cancer is the third most prevalent malignancy among women worldwide [1], with an estimated approximately 530000 new cases and 275,000 women death each year.

Early-stage patients (I–IIA) can get a satisfying outcome through radical surgery or radiotherapy, with an overall 5-year survival of >65%. Nevertheless, patients with advanced stage (IIB–IV) can only be treated with radiotherapy or plus chemotherapy, the 5-year survival rate for patients with stage III is 25 to 35%, but for stage IV is 15% or fewer [2], [3]. There are several high risk factors are thought to be closely associated with unfavorable clinical outcome, including advanced International Federation of Obstetrics and Gynecology (FIGO) stage, large tumor size, lymph node metastasis, deep cervical stromal invasion and lymphovascular space invasion. Patients with the high risk factors always develop resistance to chemotherapy and radiotherapy, finally died of local recurrence or distant metastasis. Therefore, there is an urgent need to look for novel biomarkers as a complementary predictive indicator for early diagnosis and accurate prognosis assessment, which would be helpful in targeting therapies of cervical cancer.

Trophoblast cell surface antigen 2 (TROP2) is a 36 kDa transmembrane glycoprotein belonging to tumor-associated calcium signal transducer (TACSTD) gene family. It was originally identified in human trophoblast cell lines, and elevated expression was found in various types of epithelial carcinomas while low or restricted expression was found in normal tissues [4]. Besides TROP2, epithelial cell adhesion molecule (EpCAM) gene is the another highly conserved member of TACSTD gene family, they share 49% sequence homology with both thyroglobulin type I and interleukin-2 receptors [5]. Although the regulation of expression of TROP2 gene is not fully understood, the phosphorylation sites of the cytoplasmic tail region and a conserved tyrosine and serine phosphorylation site are considered to play an important role in signal transduction. Early studies found that cross-linking TROP2 with antibodies result in the cytoplasmic calcium [Ca^2+^] increased by three times than the basal level, which suggested a mobilization of Ca^2+^ from internal stores [6]. When phosphatidylinositol 4, 5-bis phosphate (PIP2) binding to the cytoplasmic tail of TROP2, it could potentially result in an increase of inositol 1,4,5-triphosphate (IP3), which is essential for Ca^2+^ mobilization. With more Ca^2+^ released from the endoplasmic reticulum, protein kinase C (PKC) could be activated in a positive feedback mechanism which could in turn lead to the phosphorylation of more TROP2, this process could have a significant effect on the activation of the Raf, MAPK and NF-κB pathways and so on [7].

Recent work demonstrated that TROP2 behaved as a true oncogene leading to the tumorigenesis and invasiveness in colorectal cancer cell lines [8], and the overexpression of TROP2 was closely associated with cancer progression and poor prognosis. Researchers have found that bicistronic cyclin D1-TROP2 mRNA was frequently expressed in ovarian, colonic and endometrial cancers, and both the TROP2 and cyclin D1 moieties in the chimera could induce cell malignant transformation [9]. These findings all indicate that TROP2 is not only a potential prognosis biomarker, but also candidate as a therapeutic target which could be employed in developing innovative treatment strategies. In present study, we investigated TROP2 protein expression and its correlation with clinicopathologic features and clinical outcomes in cervical cancer samples. In addition, we assessed the effects of TROP2 expression on the proliferation, cell cycle and invasion in four cervical cancer cell lines, we also determined whether TROP2 plays a role in the chemotherapy of cervical cancer. These data might provide information for the prediction of cervical cancer prognosis and the establishment of targeted therapies.

## Methods

### Clinicopathologic Information of Cervical Cancer Patients

A total of 160 samples obtained by punch biopsy, cone biopsy or hysterectomy were retrieved from the Department of Pathology, Qilu Hospital of Shandong University between April 2005 to October 2007 (Table 1). The study included 106 patients with cervical cancer (mean age 43.6±11.5 years), 34 patients with cervical intraepithelial neoplasia (CIN) (mean age 40.2±8.1 years) and 20 patients with normal cervical tissues (mean age 46.4±4.8 years). Normal cervical tissue samples were obtained from patients who underwent hysterectomy for benign leiomyoma. All of the enrolled participants had intact follow-up information and were not exposed to radiotherapy or chemotherapy prior to surgical resection. The specimens were histopathologically confirmed by three pathologists according to the diagnostic criteria. Clinical stage of cervical cancer was classified according to the International Federation of Gynecology and Obstetrics (FIGO) criteria. During the follow-up period (median follow-up, 60 months; range 9.6–82.5 months), 68 patients (64.15%) were alive and 38 patients (35.85%) were dead. This study was reviewed and approved by the Institutional Medical Ethics Committee of Qilu Hospital, Shandong University. All the participants provided their written informed consent to be involved in this work.

**Table 1:** Patients characteristics and TROP2 expression.

Characteristic	Total				TROP2 expression				p	
			−		+	++	+++			
**Normal**		20	11 (55.0%)		8 (40%)	1 (5%)	0 (0%)			0.000
**CIN**		34	12 (35.3%)		10 (29.4%)	4 (11.8%)	8 (23.5%)			
**Cervical cancer**		106	12 (11.3%)		30 (28.3%)	32 (30.2%)	32 (30.2%)			
**CIN**								0.037		
CINI		12	6 (50.0%)		5 (41.7%)	1 (8.3%)	0 (0%)			
CINII		9	3 (33.3%)		4 (44.4%)	1 (11.1%)	1 (11.1%)			
CINIII		13	3 (23.1%)		1 (7.7%)	2 (15.4%)	7 (53.8%)			
**TROP2 expression in cervical cancer**										
**Age (years)**									0.100	
≤50		43	6 (14%)	9 (20.9%)		10 (23.3%)	18 (41.9%)			
>50		63	6 (9.5%)	21 (33.3%)		22 (34.9%)	14 (22.2%)			
**Histological type**									0.023	
Squamous		77	6 (7.8%)	18 (23.4%)		28 (36.4%)	25 (32.5%)			
Adenocarcinoma		29	6 (20.7%)	12 (41.4%)		4 (13.8%)	7 (24.1%)			
**FIGO stage**									0.000	
I		41	10 (24.4%)	12 (29.3%)		16 (39.0%)	3 (7.3%)			
II		59	2 (3.4%)	18 (30.5%)		14 (23.7%)	25 (42.4%)			
III and IV		6	0 (0%)	0 (0%)		2 (33.3%)	4 (66.7%)			
**Histological grade**									0.000	
Well		35	7 (20.0%)	17 (48.6%)		5 (14.3%)	6 (17.1%)			
Moderate		33	4 (12.1%)	8 (24.2%)		15 (45.5%)	6 (18.2%)			
Poorly		38	1 (2.6%)	5 (13.2%)		12 (31.6%)	20 (52.6%)			
**Lymphatic metastasis**									0.001	
NO		79	12 (15.2%)	27 (34.2%)		23 (29.1%)	17 (21.5%)			
YES		27	0 (0%)	3 (11.1%)		9 (33.3%)	15 (55.6%)			
**Invasive interstitial depth**									0.000	
<1/2		53	12 (22.6%)	21 (39.6%)		18 (34.0%)	2 (3.8%)			
≥1/2		53	0 (0%)	9 (17%)		14 (26.4%)	30 (56.6%)			
**Tumor size**									0.255	
d≤4 cm		86	9 (10.5%)	25 (29.1%)		29 (33.7%)	23 (26.7%)			
d>4 cm		20	3 (15.0%)	5 (25.0%)		3 (15.0%)	9 (45.0%)			

### Immunohistochemistry

Immunohistochemical staining was carried out on 4-µm thick sections of the paraffin-embedded tissues, and the staining process was strictly performed according to the streptavidin-biotin-peroxidase complex method. After deparaffinisation and rehydration, the sections were treated with 3% hydrogen peroxide to block endogenous peroxidase. Non-specific binding was blocked with normal goat serum for 30 min at 37°C, then the sections were incubated with anti-TROP2 (Santa Cruz, USA, 1∶500 dilution) or anti-Ki-67 monoclonal antibody (Dako, lostrup, Denmark, 1∶100 dilution) overnight at 4°C. After washing with PBS, the sections were incubated with a horseradish peroxidase-labeled polymer-conjugated anti-mouse secondary antibody (Beijing Zhong Shan Biotech Co. Ltd, BeiJing, China) at 37°C for 30 min. Then the sections were stained with 3,3-diaminobenzidine tetrahydrochloride for 5 min and nuclei were counterstained with hematoxylin for 3 min, and then mounted with neutral balsam. PBS was used to replace the antibody as a negative control.

### Evaluation and Quantification of Immunostaining

The immunostaining results were evaluated by three pathologists who were blinded to the clinical details of patients. TROP2 expression was defined as the presence of yellow-brown membrane staining of tumor cells. Each sample should be estimated including the staining intensity and percentage of positive tumor cells with no less than 1000 cells and 5 high power fields. The intensity of staining was scored as 0 (negative), 1 (weak), 2 (moderate) and 3 (strong), while the percentage of positive cells was scored as 0 (0%), 1 (1–10%), 2(11–50%) and 3 (51–100%). The overall immunohistochemical staining results were based on the intensity score×percentage staining score as follows: − (score 0); +(score 1, 2, 3); ++(score 4, 6); +++(score 9) [10]. For Ki-67, the labeling index (LI) was assessed as the percentage of cells with nuclear staining among 1,000 invasive tumor cells in randomly selected, the final score of Ki-67 expression was categorized into four groups based on the labeling index and location of nuclear staining as described previously [11]. The classification was as follows: −, (<10%, restricted to the parabasal cell layers); +, (10% to <30%, restricted to the lower third of the epithelium); ++, (30 to <70%, reaching the upper third of the epithelium); +++, (70% or more of the epithelial cells including full thickness express ion of Ki-67).

### Cell Culture

Four human cervical cancer cell lines CaSki, Siha, HeLa and C33A cells were purchased from the American Type Culture Collection (Manassas, VA, USA), cultured in Dulbecco-modified Eagle medium (DMEM; Gibco Inc., Carlsbad, CA, USA) containing 10% fetal bovine serum (Invitrogen, Carlsbad, CA, USA) and 1% penicillin-streptomycin (Invitrogen) in a humidified incubator at 37°C and 5% CO_2_ atmosphere.

### Immunofluorescence Staining

Siha and CaSki cells were cultured on glass slides in a 6-well plate and incubated for 24 h. Cells were fixed with 4% paraformaldehyde and blocked with normal goat’s serum for 30 min at 37°C. After thorough washing with Tris-buffered saline (TBS), the cells were incubated with primary anti-TROP2 monoclonal antibody (Santa Cruz, USA, 1∶500 dilution) overnight at 4°C and stained with FITC conjugated anti-mouse IgG (Beijing Zhong Shan Biotech Co. Ltd, BeiJing, China, 1∶200 dilution) for 30 min. The fluorescence-labeled TROP2 was observed under a fluorescence microscope and photograph was taken.

### Cells Transfections

To knock down endogenous TROP2 expression, two pairs of siRNA sequences targeting TROP2 were designed and synthesized by Genepharma Co., Ltd (Shanghai, China). These sequences were: siRNA-1100, 5′-GCACGCUCAUCUAUUACCUTT-3′, 5′-AGGUAAUAGAUGAGCGUGCTT-3; siRNA-550, 5′-CCAAGUGUCUGCUGCUCAATT-3′, 5′-UUGAGCAGCAGACACUUGGTT-3′. Negative scramble control sequences were: 5′-UUCUCCGAACGUGUCACGUTT-3′, 5′-ACGUGACACGUUCGGAGAATT-3′. To study the effects of TROP2 enforced expression, the TROP2 isoform expressing plasmid was employed in HeLa and C33A cells. The human TROP2 full length cDNA was amplified and inserted into the pcDNA 3.1 vector (Genepharma Co., Ltd Shanghai, China) to obtain pcDNA3.1-TROP2. For transfection, cells were seeded into 6-well plates and expected to be 50% confluency next day. After cell attachment, TROP2 siRNA or the recombinant pcDNA3.1-TROP2 were transfected into cells in Opti-MEM (Invitrogen) using the Lipofectamime 2000 transfection reagent (Invitrogen) according to the manufacturer’s instruction, culture medium was replaced after 6 hours of incubation. After 48 h transfection, cells were counted and subjected to cell viability assay and western blot analysis. Untransfected cells were thought to be blank control, cells transfected with scrambled siRNA or pcDNA3.1(empty vector) were considered as negative control.

### Cell Viability Assay

Cell viability was determined using a cell counting kit-8 (CCK-8) in accordance with the manufacturer’s protocol (Jingmei biotech, Shanghai, China). Briefly, control and transfected cells were seeded at 5000 per well in 96-well plates, after treated with cisplatin (Sigma, St. Louis, MO, USA) or MEK inhibitor U0126 (Cell Signaling Technology, Beverly, MA, USA) at indicated time points, 10 ul of CCK-8 was added to each well, then incubated for an additional 2 h at 37°C. The optical density (OD) was measured using a microplate reader (Bio-Rad Model 680, Richmond, CA, USA) at 450 nm wavelength. The inhibitory concentrations of 50% proliferation (IC50) of cisplatin were calculated by GraphPad Prism software. The experiment was repeated three times.

### Apoptosis Assay

At 48 h after transfection, the cells were collected and washed twice with cold PBS, resuspended in 400 uL Annexin V-FITC binding buffer at a density of 1×10^6^ cells/ml. Cells were stained with 5 uL of Annexin V-FITC and 10 ul propidium iodide (PI) according to the Apoptosis Detection Kit (Jingmei biotech, Shanghai, China) instructions. Then subjected to flow cytometry (BD, San Jose, CA, USA) to detect cell apoptosis. This experiment was conducted three times.

### Cell Cycle Analysis

Cells transfected with TROP2 siRNA or pcDNA3.1-TROP2 were harvested at 48 h after transfection, collected by trypsinization and fixed in 75% cold ethanol for 1 h at −20°C. After being washed with PBS, cells were incubated with 100 µl RNase A (100 µg/mL) and 400 µl propidium iodide respectively for 30 min at 37°C. Finally, the cell cycle was measured by flow cytometry using a FACScan flow cytometer (BD, San Jose, CA, USA) at 488 nm, and the relative ratios of the G1, S, and G2 phases was analyzed by FlowJo 2.8 software. The experiment was performed in triplicate.

### Wound Healing Assay

The monolayer wound healing assay was used to assess cell migration ability. Cells (5×10^5^) were seeded in 6-well plates, incubated overnight, then transfected with TROP2 siRNA or pcDNA3.1-TROP2. After achieving 90% confluency, the cell monolayer was scratched with a sterile pipette tip, floating cells were removed with PBS and cultured again in RPMI 1640 medium containing 1% FBS. Photographic images were taken at 0, 24 and 48 h along the scrape line by microscope. Results were expressed as relative scratch width, based the distance migrated relative to the original scratched distance. The experiment was conducted in triplicate.

### Cell Invasion Assay

The invasive ability of cervical cancer cells was assessed using a 24-well transwell chamber (cell invasion assay kit), by calculating the cells passed through a polycarbonate membrane (8-µm pore size) (Corning Costar, New York, USA). The polycarbonate surface of each chamber was covered with 20 uL matrigel (BD Biosciences, USA; 1∶4 dilution) to create an artificial basement membrane. Cells (1×10^5^ cells) transfected with TROP2 siRNA or pcDNA3.1-TROP2 were suspended in 200 ul serum-free 1640 medium and cultured in the upper transwell chamber for 24 h at 37°C, the lower chamber was filled with 600 uL of 1640 medium supplemented with 10% FBS. The non-invading cells attached to the upper surface of the membrane were removed with a sterile cotton swab, and the invasion cells penetrated through the membrane were stained with 0.1% crystal violet for 20 min at room temperature. The numbers of cells were calculated under a Leica microscope in eight random fields. The experiment was repeated three times for each group.

### Hoechst 33258 Staining

At 24 h after transfection, the transfected cells and control cells were exposed to different concentrations of cisplatin for 48 h. Then the cells were fixed with 4% paraformaldehyde for 15 minutes followed by staining with 100 µg/ml of Hoechst 33258 (Beyotime, Shanghai, China) at room temperature in the dark for 30 minutes, the apoptotic features were assessed by observing chromatin condensation or fragments under a fluorescence microscope with an excitation wavelength of 340 to 360 nm.

### Western Blot Analysis

Equivalent amount of the cell protein samples were loaded on 10% sodium dodecyl sulfate polyacrylamide gel electrophoresis (SDS–PAGE) and transferred to PVDF membranes using the Bio-Rad electrotransfer system. After blocked by 5% w/v nonfat dried milk for 1 h, the membranes were incubated with primary antibodies specific to TROP2 (Santa Cruz Biotechnolog, CA, USA; 1∶1000 dilution), E-cadherin, cyclin D1, cyclin E, CDK2, CDK4, Erk1/2, p-Erk1/2, P27, bcl-2 and bax (Cell Signaling Technology, Beverly, MA, USA; 1∶1000 dilution) overnight at 4°C. Then incubated with horseradish peroxidase-conjugated rabbit anti-mouse secondary antibody (Beijing Zhong Shan Biotech Co. Ltd, BeiJing, China; 1∶4000 dilution ) for 1 h, the protein bands were visualized by enhanced chemiluminescence (Millipore) and analyzed densitometrically using Quantity One Image software (Bio-Rad, USA). β-actin (Beijing Zhong Shan Biotech Co. Ltd, Beijing, China; 1∶1000 dilution) was used as internal control for protein loading and analysis.

### Statistical Analyses

SPSS (SPSS Inc, Chicago, IL, USA) 18.0 software was used to perform statistical analysis.

Quantitative data were expressed as the mean values ± standard deviation (SD). Differences between groups were evaluated by unpaired Student’s t test or one-way ANOVA. Pearson chi-square test or Fisher’s exact test was used for analysis between the degree of staining and clinical parameters, correlation analysis was performed with Spearman rank correlation coefficient. Kaplan–Meier curves were plotted to describe the survival information by the log-rank test. Multivariate analysis was performed using a Cox’s proportional hazards regression model. P<0.05 was considered statistically significant.

## Results

### TROP2 is Over-expressed in Highly Proliferative Cervical Cancers

**Figure 1:**
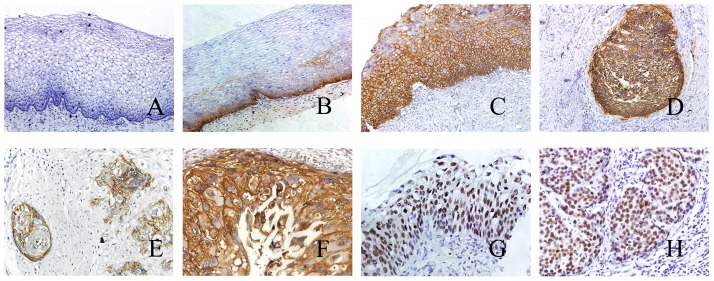
Immunohistochemical staining of TROP2 and Ki-67 in cervical tissues. A–B. Negative and weak TROP2 expression in normal cervical tissues. C. CIN III with strong TROP2 membrane staining (score +++). D–E. Strong and weak TROP2 expression in cervical sqamous cell carcinoma (score +++, +). F. Strong TROP2 expression in cervical adenocarcinoma (score +++). G–H. Ki-67 expression in CINIII and cervical cancer (score ++, +++). Magnifications: ×200 (A, B, C, D, E) and ×400 (F, G, H).

In total, 160 samples were determined by immunohistochemistry, including 20 normal cervical tissues, 34 CIN tissues and 106 cervical cancer tissues. Demographic data and tumor characteristics are described in Table 1. None of the patients had received radiotherapy or chemotherapy prior to surgery. According to criteria defined earlier, 55% of normal cervical specimens showed undetectable TROP2 expression, 40% and 5% of samples displayed weak and moderate staining, predominantly detected in ectocervical squamous epithelium (Figure 1A and B). Intriguingly, the expression of TROP2 was gradually increased from CINI (50%) to CINII (66.7%) and CINIII (76.9%; Figure 1C) (p = 0.037), indicating TROP2 expression was significantly correlated with the development and progression of cervical cancer. Moreover, intense membranous staining of TROP2 was detected in majority (88.7%) of cervical cancer cases while stromal cells were regularly negative (Figure 1D, E and F). Statistical analysis showed that TROP2 expression level in cervical cancer cases was significantly higher than that in normal cervical tissues and CIN tissues (p<0.001).

For the sake of verifying the association between TROP2 expression and cancer progression, we also analyzed the expression of Ki-67 in the same sets of cervix specimens by immunohistochemistry assay (Figure 1G and H). Ki-67 has proven to be a common proliferative marker and used to evaluate many malignant lesions of cancers. Statistical analysis revealed that there was a significant correlation between increased TROP2 staining and Ki67-positive proliferating cells in cervical cancer specimens (p<0.001; Table 2), which indicated that TROP2 was over-expressed in highly proliferative human cervical cancer cells.

**Table 2:** Ki-67 expression and the correlation with TROP2.

Ki-67 expression										
		Total		−		+		++	+++	p
**Normal** **tissue**		20		15 (75.0%)		3 (15.0%)		2 (10.0%)	0 (0%)	<0.001
**CIN**		34		15 (44.1%)		6 (17.6%)		7 (20.6%)	6 (17.6%)	
**Cervical** **cancer**		106		22 (20.7%)		22 (20.7%)		25 (23.6%)	37 (34.9%)	
**Ki-67 expression in cervical cancer**										
**TROP2 expression**			**−**		**+**		**++**		**+++**	**p**
−	12		7 (58.3%)		4 (33.3%)		1 (8.3%)		0 (0%)	<0.001
+	30		8 (26.7%)		6 (20%)		9 (30%)		7 (23.3%)	
++	32		6 (18.7%)		4 (12.5%)		7 (21.9%)		15 (46.8%)	
+++	32		1 (3.1%)		8 (25%)		8 (25%)		15 (46.8%)	

### Correlation between TROP2 Expression and Clinical Pathological Features

The clinical significances of TROP2 overexpression in patients with cervical cancer were summarized in Table 1. The results showed that TROP2 expression levels were significantly different between groups in regards to histological grade (p<0.001), lymphatic metastasis (p = 0.001), invasive interstitial depth (p<0.001), FIGO stage (p<0.001) and histological-subtype (p = 0.023). However, there was no significant associations between TROP2 expression and age (p = 0.100), tumor size (p = 0.255).

### High Expression of TROP2 Correlates with Poor Prognosis in Cervical Cancer

**Figure 2:**
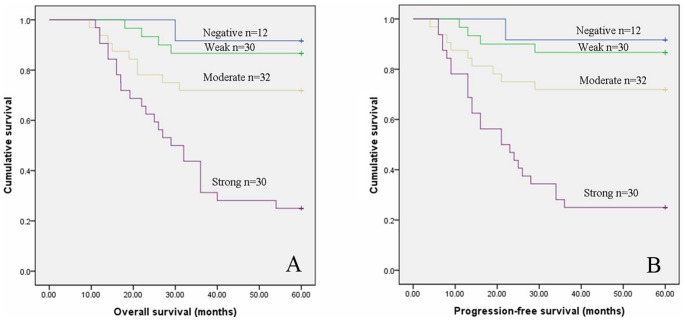
Kaplan- Meier survival curves of patients with cervical cancer according to TROP2 expression. A. Overall survival (OS). B. Progression-free survival (PFS).

To evaluate the impact of TROP2 expression and clinicopathological features on clinical outcomes of the patients, we used Kaplan -Meier analysis and the log-rank test for censored survival data. As displayed in Figure 2, Kaplan–Meier survival curves showed that patients with positive TROP2 expression had poorer overall survival (OS) compared to those without TROP2 expression (p<0.01), progression-free survival (PFS) also gradually declined with increasing TROP2 expression scores (p<0.01). By univariate analysis we found that clinicopathologic characteristics including advanced FIGO stages, poorly histological grade, positive lymphatic metastasis and deeper invasive interstitial depth had a significantly inferior clinical outcome (Table 3). Next, the expression of TROP2 as well as all the statistically significant variables evaluated in the univariate analyses were examined by multivariate Cox regression model analysis. TROP2 protein overexpression, along with histological grade and lymphatic metastasis were identified to be independent predictive factors for poor OS (p = 0.022, p = 0,023 and p = 0.002, respectively) and PFS (p = 0.016, p = 0.04 and p = 0.005, respectively).

**Table 3:** Univariate and multivariate analyses of OS and PFS in relation to clinical parameters and TROP2 expression.

Parameter	Univariate analysis				Multivariate analysis							
	p^a^		p^b^	p^a^	RR	95% CI	p^b^	RR			95% CI	
**TROP2 expression**	0.000	0.000		0.022	1.891	1.097–3.261	0.016	1.952		1.136–3.357		
(Positive vs Negative)												
** Age**	0. 427	0.402										
(>50 vs ≤50)												
** Histological type**	0.583	0.578										
(Squamous vs Adenocarcinoma)												
**FIGO stage**	0.001	0.001		0.616	1.194	0.598–2.383	0.599	1.208	0.597–2.444			
(I vs II vs III–IV)												
**Histological grade**	0.000	0.000		0.023	1.804	1.084–2.999	0.040	1.706	1.026–2.838			
(well vs moderately vs poorly)												
**Lymphatic metastasis**	0.000	0.000		0.002	0.343	0.173–0.678	0.005	0.375	0.190–0.743			
(yes vs no)												
**Invasive interstitial depth**	0.000	0.000		0.547	0.759	0.309–1.863	0.535	0.753	0.307–1.844			
(<1/2 vs ≥1/2)												
**Tumor size**	0.211	0.201										
(≤4 cm vs >4 cm)												

a Overall survival(OS).

b Progression-free survival (PFS).

RR: relative risk.

### TROP2 Promotes Cell Proliferation in Cervical Cancer Cells

**Figure 3:**
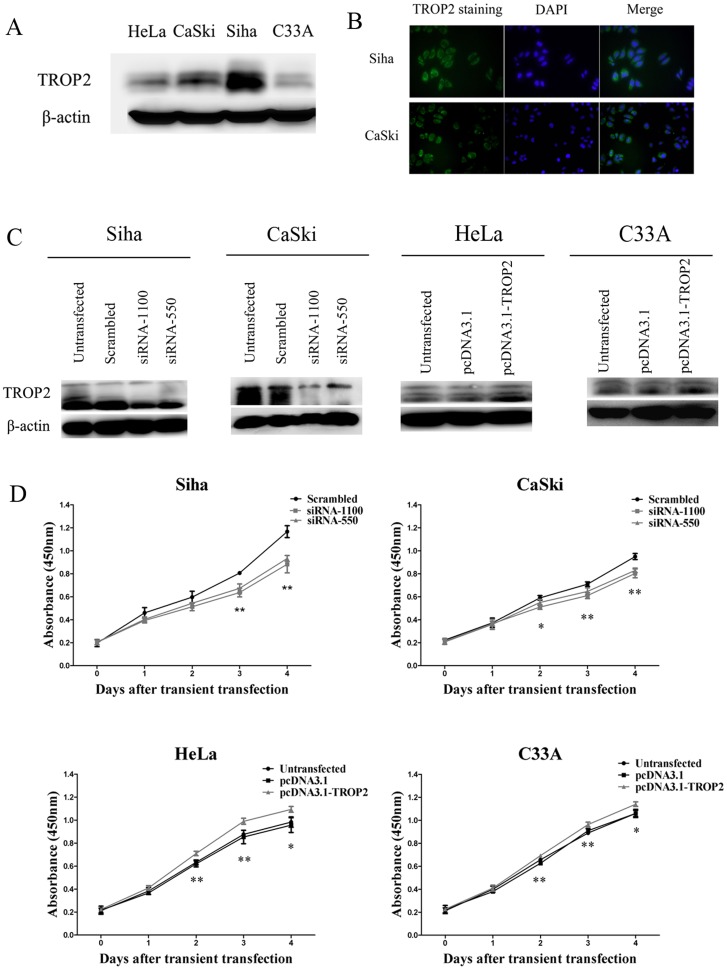
The expression of TROP2 in cervical cancer cell lines and its effects on cell proliferation. A. The expression of TROP2 was found in four cervical cancer cell lines by western blot, Siha and CaSki cells showed higher expression compared with HeLa and C33A cells, β-actin was used as internal control. B. Immunofluorescence assay demonstrated there was a layer of green fluorescent staining of the cytomembrane in Siha and CaSki cells. C. Western blot analysis to confirm the siRNA mediated knockdown and pcDNA3.1-TROP2 mediated overexpression of TROP2 in different cervical cancer cell lines. D. Cells were transfected with TROP2 siRNA or pcDNA3.1-TROP2, the viability was assessed using a CCK-8 assay at five time points (0, 1, 2, 3, 4 days, respectively). At 2 days after transfection, knockdown of TROP2 elicited a significant inhibit effect on cell proliferation in Siha and CaSki cells, while enforced expression of TROP2 increased cell viability of HeLa and C33A cells. Data are expressed as mean ± SD of three independent experiments, *p<0.05, **p<0.01.

Given the evidence that TROP2 elevated expression was closely associated with tumor aggressiveness and poor clinical prognosis, we further investigated the functional consequences of TROP2 expression in cervical cancer cell lines. First of all, we examined the TROP2 expression in four cervical cancer cell lines Siha, HeLa and CaSki and C33A by Western blot (Figure 3A). Consistent with the immunohistochemistry assay, TROP2 protein was evidently detected in all cell lines at about 36 kDa, Siha and CaSki cells showed relatively higher TROP2 expression, whereas its expression was weak in HeLa and C33A cells. By immunofluorescence analysis we found that there was a layer of green fluorescent staining of the cytomembrane of Siha and CaSki cells (Figure 3B). To further explore the biological functions of TROP2, we identified and validated two independent and non-overlapping siRNA sequences to deplete endogenous TROP2 expression in Siha and CaSki cells, HeLa and C33A cells were transfected with pcDNA3.1-TROP2 to enhance TROP2 expression. Cells transfected with scrambled siRNA or pcDNA3.1 were considered as negative control, non-transfected cells were used as blank control. According to the results of Western blot at 48 h post-transfection (Figure 3C), the protein level of TROP2 displayed a significant variation, the two siRNA sequences all lead to reduced TROP2 expression by 50%–80% of Siha and CaSki cells, and pcDNA3.1-TROP2 was able to produce at least 30% up-regulation of TROP2 expression of HeLa and C33A cells.

CCK-8 assay was used to determine the effect of TROP2 expression on cell proliferation. Our results revealed that knockdown of TROP2 evoked a markedly inhibition effect on the proliferation after transfection in Siha and CaSki cells (p<0.05), whereas the cell viability in the pcDNA3.1-TROP2 transfection group was improved significantly (p<0.05; Figure 3D). Combined with our immunohistochemistry results showed that TROP2 was highly expressed in Ki67 positive proliferating cells, these data indicated that the expression of TROP2 increased the cell viability of cervical cancer cells.

### Impact of TROP2 Expression on Cell Apoptosis and Cell Cycle

**Figure 4:**
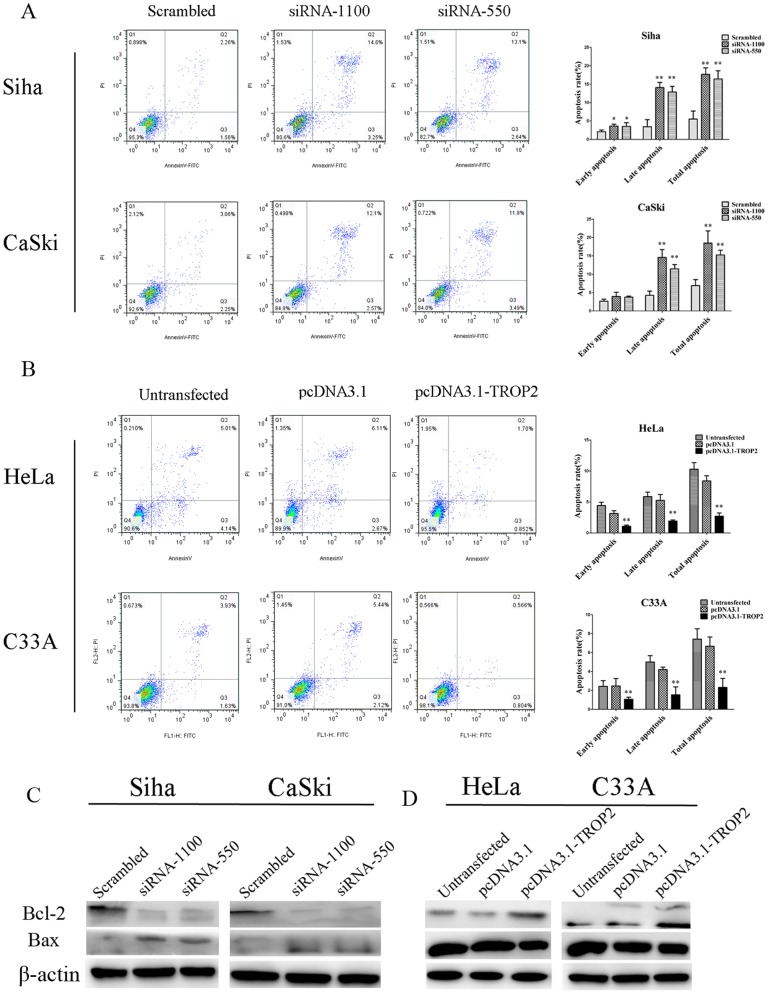
The impact of TROP2 expression on cell apoptosis. A. Down-regulation of TROP2 induced cell apoptosis in Siha and CaSki cells. Cells were harvested at 48 h after transfection, followed by apoptosis assay using the annexin V-FITC apoptosis detection kit. Cells in the right lower and upper quadrants are consider as early and late apoptosis respectively, in the left upper quadrants are considered as dead cells. The results were analyzed by FlowJo software. B. Enforced expression of TROP2 significantly inhibited cell apoptosis C. Western blot analysis showed that down-regulation of TROP2 reduced the expression of bcl-2 and increased the expression of bax in Siha and CaSki cells. D. Enforced expression of TROP2 in HeLa and C33A cells increased the expression of bcl-2 and reduced the expression bax. These data are expressed as mean ± SD of three independent experiments, *p<0.05, **P<0.01.

It has been demonstrated that cell apoptosis plays a considerable role in the progression and development of tumors, we further explored whether the cell proliferation inhibition due to the apoptosis-induced function. Flow cytometry was used to determine cell apoptosis at 48 h after transfection. The results showed that the total cell apoptosis rates of the TROP2 siRNA transfection group (siRNA-1100 and siRNA-550) was significantly higher compared with negative control group in both Siha and CaSki cells (p<0.05; Figure 4A). Meanwhile, the tendency of early cell apoptosis and late cell apoptosis rates was consistent with that of total cell apoptosis rates. On the contrary, enforced expression of TROP2 significantly inhibited cell apoptosis, the proportions of positive cells for annexin V and propidium iodide were evidently decreased in HeLa and C33A cells, compared to untreated cells and pcDNA3.1 transfected cells (P<0.01, respectively; Figure 4B). To further explore the underlying molecular mechanisms by which TROP2 knockdown induces apoptosis, we investigated the expressions of bcl-2 and bax by Western blot (Figure 4C). The results showed that the expression of bcl-2 protein was obviously decreased while bax was markedly enhanced in both Siha and CaSki transfected cells compared to control cells (scrambled siRNA). In contrast, HeLa and C33A cells transfected with pcDNA3. 1-TROP2 exhibited the opposite effect (Figure 4D). These findings demonstrated that anti-apoptotic effect of TROP2 is achieved by directly upregulating bcl-2 expression and inhibiting bax activation.

**Figure 5:**
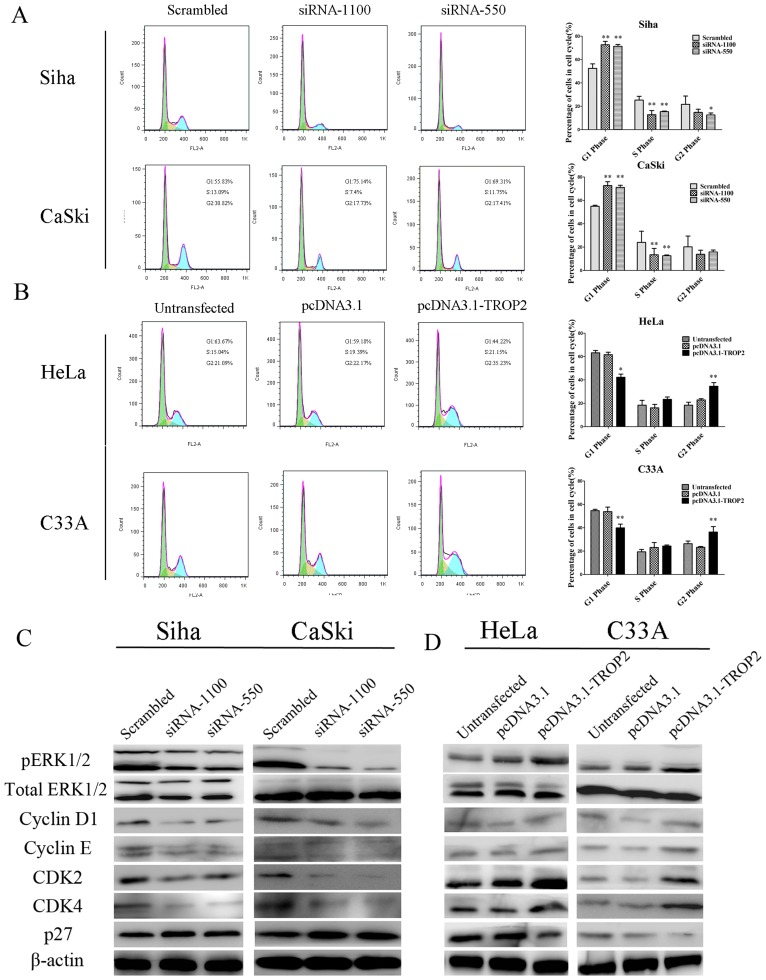
The effect of TROP2 expression on cell cycle. A. Knockdown of TROP2 induced G1 arrest in Siha and CaSki cells. Siha and CaSki cells were transfected with TROP2 siRNA or scrambled siRNA. 48-regulation of TROP2 induced cell cycle arrest in G1 phase. B. Overexpression of TROP2 promoted G1-S and G2-M transitions in HeLa and C33A cells. C, D. TROP2 regulated cell cycle factors via ERK1/2 signaling pathway in cervical cancer cells. Siha and CaSki cells (C) were transfected with TROP2 siRNA or scrambled siRNA. 48 h later, Western blot analysis showed that the changes in cell cycle distribution was accompanied by reduced expression of cyclin D1, cyclin E, CDK4, CDK2 and increased expression p27. Knockdown of TROP2 also inhibited the phosphorylation of ERK1/2. HeLa and C33A cells (D) were transfected with pcDNA3.1-TROP2, and 48 h post transfection, protein were extracted and subjected to Western blot analysis of related proteins. Data are expressed as mean ± SD of three independent experiments, *p<0.05, **P<0.01.

To obtain a more comprehensive understanding of TROP2 gene function, we further investigated the effects of TROP2 expression on the cell cycle by flow cytometry analysis. As shown in Figure 5A, the results indicated that depletion of TROP2 expression results in the accumulation of cells in G1 phase and the reduction of cells in S phase at 48 h after transfection (p<0.05). In contrast, enforced expression of TROP2 in HeLa and C33A cells showed a significant reduction of cells in the G1 phase but an increase of cells in the S phase and the G2-M phase (p<0.05; Figure 5B). The results indicated that TROP2 was capable of enhancing cervical cancer cells proliferation by promoting G1-S and G2-M transitions.

### Effect of ERK1/2 Signaling Pathway on the Expression of TROP2 in Cervical Cancer Cells

ERK1/2 (extracellular signal regulated kinase) is a key member of MAPK (mitogen-activated protein kinases) signaling pathway, can be activated by a variety of stimuli, the posphorylation of ERK1/2 is involved in a lot of biological behaviors, such as cell proliferation, cell cycle, adhesion and invasion, as well as survival and metastasis. Recent studies have demonstrated that an involvement of TROP2 in MAPK signaling pathway, so we hypothesized that the posphorylation of ERK1/2 may also contribute to the changes of cell cycle mediated by TROP2 in cervical cancer. As shown in Figure 5C, at 48 h after transfection, down-regulation of TROP2 exhibited substantially decreased levels of pERK1/2 expression in both Siha and CaSki cells, in contrast, pERK1/2 protein level was upregulated in HeLa and C33A cells which transfected with pcDNA3.1-TROP2 (Figure 5D), while the total ERK1/2 expression was unaffected. It is well documented that ERK1/2 MAPK signaling pathway generates a multitude of intracellular responses through a plethora of downstream molecules. Cyclin D1 and cyclin E are downstream targets of the ERK1/2 pathway, and the high expression could prevent cell cycle arrest in the G0/G1 phase, accelerate S phase progression. CDK2 and CDK4 are two important cyclin-dependent kinases, they can interact with cyclin E and cyclin D1 to form a complex which can shorten the cell cycle, leading to uncontrolled cell cycle regulation and cell proliferation. Western blot analysis indicated that the expression of these four cell cycle promoters was significantly decreased in TROP2 siRNA group, whereas the expression of cyclin-dependent kinases inhibitor p27 was increased. In contrast, enforced expression of TROP2 showed increased expression of Cyclin D1, cyclin E, CDK2 and CDK4, but decreased expression of the p27. Overall, all the data presented herein suggest that TROP2 may play a pivotal role in the regulation o f cell cycle regulators via ERK1/2 pathway.

### Effect of TROP2 Expression on Cell Migration and Invasion

**Figure 6:**
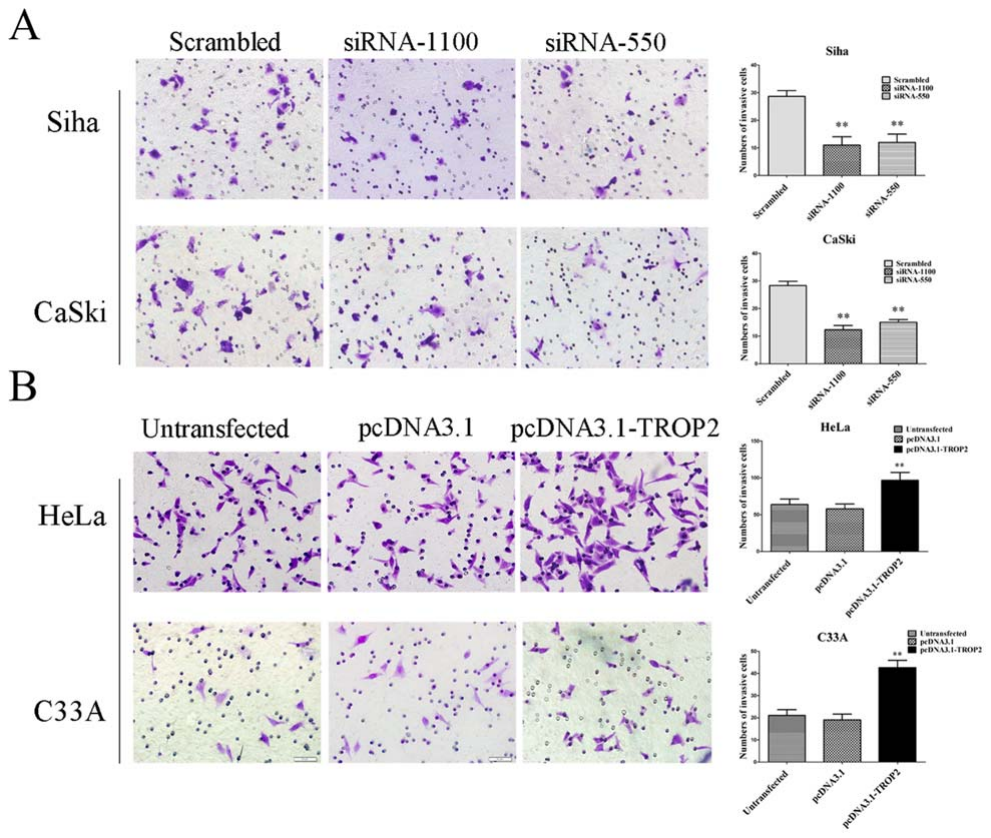
The influence of TROP2 expression on the invasive capability of cervical cancer cells. A. Down-regulation of TROP2 inhibited cell invasion of Siha and CaSki cells. At 24 h after transfection, cells (1×105) were reseeded in the upper Transwell chamber for 24 h and then stained with crystal violet. TROP2 siRNA group contained significantly less invasive cells than the control groups. B. Up-regulation of TROP2 significantly increased the invasive ability of HeLa and C33A cells at 48 h after transfection. Data are mean ± SD, *p<0.05, **P<0.01.

**Figure 7:**
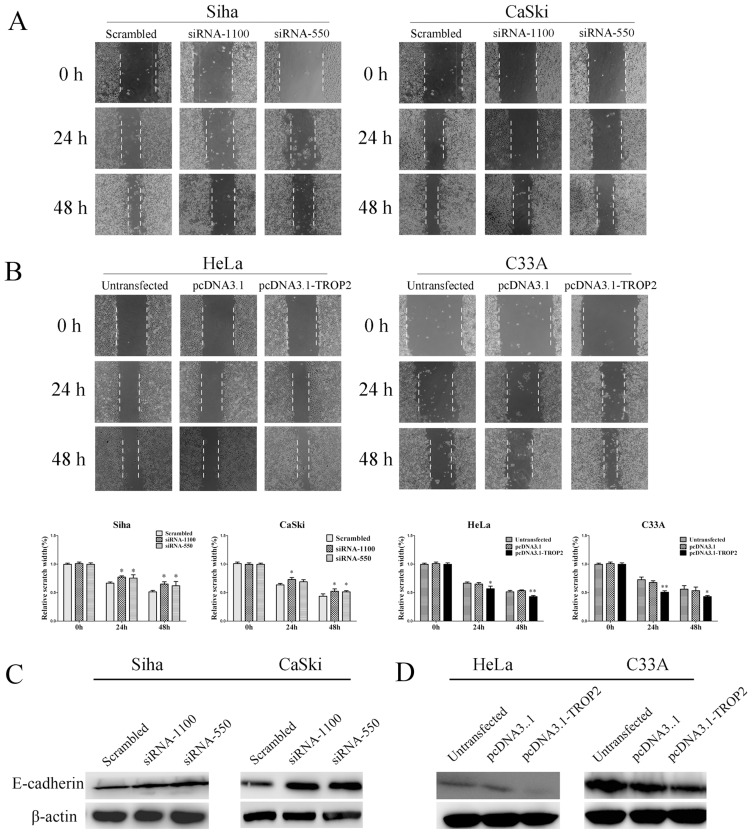
The effect of TROP2 expression on cell migration. A. Knockdown of TROP2 exhibited a slower wound recovery compared to the control groups at 24-cadherin expression of Siha and CaSki cells. D. Enforced expression of TROP2 reduced the E-cadherin expression of HeLa and C33A cells.

Our immunohistochemical analysis demonstrated that high expression of TROP2 was positively correlated with lymphatic metastasis, therefore we further tested whether TROP2 deletion would inhibit invasion and migration of cervical cancer cells. The serum-stimulated matrigel invasion assay showed that the number of Siha and CaSki cells that passed through the polycarbonat emembrane in the TROP2 siRNA transfection group was significantly less than that in the negative control group (p<0.05; Figure 6A). In contrast, up-regulation of TROP2 significantly increased the invasive ability of HeLa and C33A cells at 48 h after transfection (p<0.05; Figure 6B). Wound healing assay was used to determine the effect of TROP2 on the migration. As shown in Figure 7A, at 24 h after wounding, TROP2 siRNA transfected cells presented a significantly slower closing of scratch wound compared with control groups cells (p<0.01). On the contrary, Hela and C33A cells transfeted with pcDNA3.1-TROP2 exhibited an elevated capacity of wound healing (p<0.05, Figure 7B). As numerous studies have revealed that expression of E-cadherin is intricately linked to the tumor invasiveness, we further determined the E-cadherin expression by Western blot. We found that TROP2 siRNA transfection resulted in elevated expression of E-cadherin (Figure 7C), as expected, the enforced expression of TROP2 led to decreased expression of E-cadherin (Figure 7D). These results provided evidence for a potential role of E-cadherin during TROP2 overexpression induced proliferation and invasion in cervical cancer.

### Knockdown of TROP2 Enhances Sensitivity of Siha and CaSki Cells to Cisplatin

**Figure 8:**
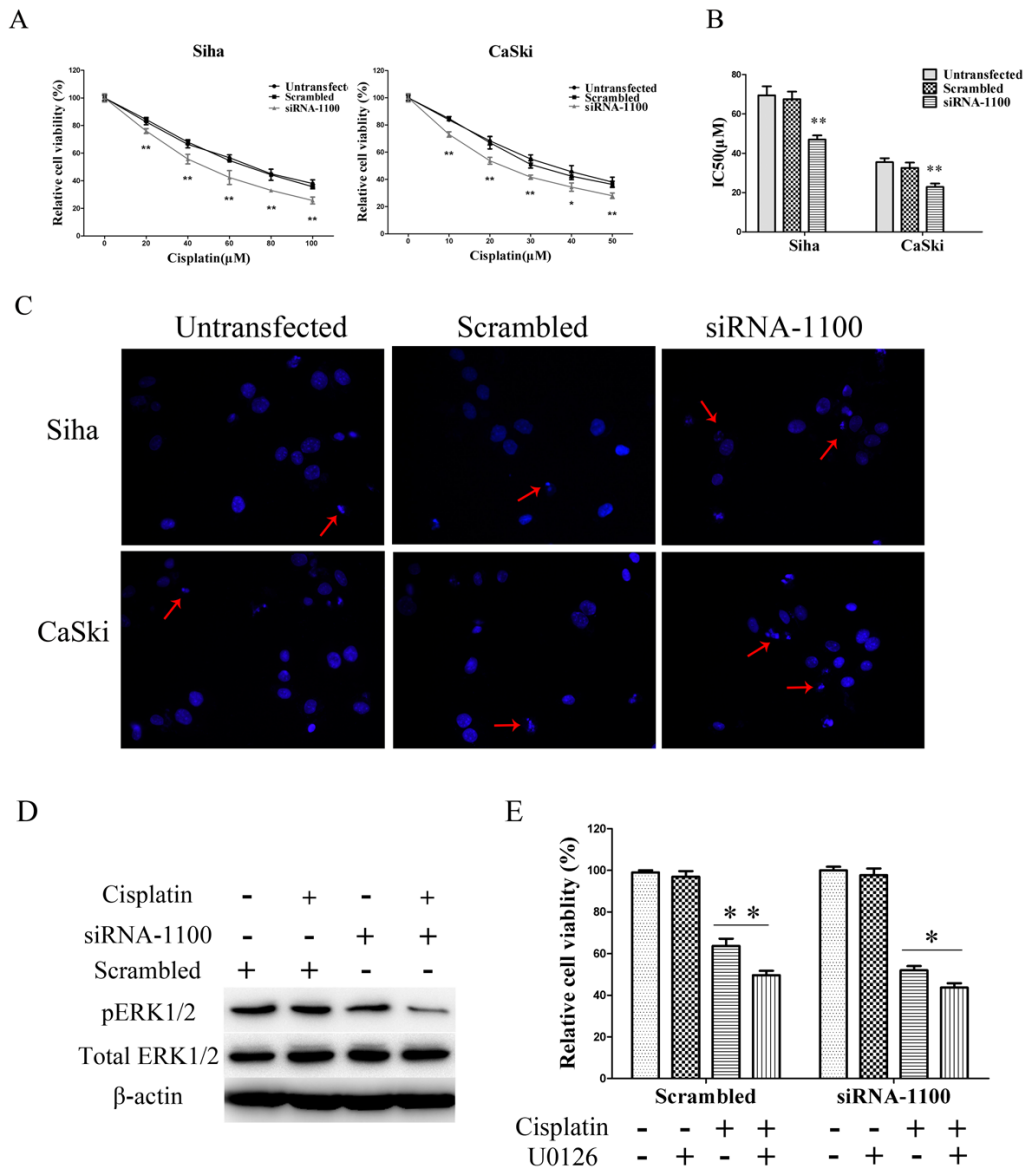
Knockdown of TROP2 sensitized Siha and CaSki cells to cisplatin. A. Effect of cisplatin on Siha and CaSki cells growth. Cells were transfected with TROP2 siRNA-1100 to deplete endogenous TROP2 expression, 24 h later, they were exposed to CDDP for another 48 h. Cell viability was determined by CCK-8 assay. B. Graphic representation of the mean IC50 values of cisplatin in Siha and CaSki cells. Cells transfected with TROP2 siRNA-1100 were more sensitive to cisplatin than control cells. C. Morphological transformation of Siha and CaSki cells exposed to cisplatin was observed under a fluorescence microscope, Hoechst 33258 staining indicated that there were more apoptotic cells in TROP2 siRNA group, compared with control groups. Red arrows indicate apoptotic cells. D. Western blot analysis showed TROP2 siRNA-1100 decreased the expression of pERK1/2 after cisplatin treatment. Siha cells were tansfected with TROP2 siRNA-1100 or scrambled siRNA, 24 h later, the cells were exposed to 30 µM cisplatin for another 48 h, and the levels of pERK1/2 was analyzed by western blot. E. ERK inhibitor U0126 enhanced TROP2 siRNA-1100 mediated cisplatin sensitivity of Siha cells. U0126 at the concentration of 10 µmol/l was used 2 h before cisplatin (30 µM) treatment. Data are presented as mean ± SD of three independent experiments, *p<0.05, **P<0.01.

Previous results showed that knockdown of TROP2 inhibited the proliferation and invasion of Siha and CaSki cells, we further investigated whether TROP2 plays a role in the chemotherapy of cervical cancer. Cisplatin is a widely used anticancer agent, has also been the primary agent used in the combination chemotherapy for cervical cancer patients. To determine the sensitivity of cells to cisplatin, at 24 h after transfection with siRNA-1100, CaSki cells were exposed to different concentration of cisplatin (CDDP) ranging from 0 to 50 µM for 48 h, as Siha cells turned out to be more resistant, they were treated with concentrations from 0 to 100 µM, the cell viability was examined by CCK-8 assay. As shown in Figure 8A, the cell survival rate appeared to show a dose -dependent manner in response to cisplatin treatment, and down-regulation of TROP2 protein resulted in enhanced sensitivity to CDDP treatment in both Siha and CaSki cells. The mean IC_50_ of Siha TROP2 siRNA-1100 transfected cells was significantly lower than untransfected and negative control cells (Figure 8B; p<0.05). CaSki cells showed the similar results (p<0.05). Based on the above results, Siha and CaSki cells were exposed to 60 and 30 µM cisplatin for 48 h, the morphological changes of apoptotic cells were visualized by Hoechst 33258 staining assay (Figure 8C). We found that the number of apoptotic cells was evidently increased in TROP2 siRNA group as compared with that of control groups. We previously demonstrated a direct link between TROP2 expression and ERK1/2 signaling pathway, so we further investigated whether down-regulation of TROP2 enhances cisplatin sensitivity of cervical cancer cells was due to the inactivation of ERK signaling pathway. Siha cells tansfected with TROP2 siRNA-1100 or scrambled siRNA were exposed to 30 µM cisplatin, and the levels of pERK1/2 was analyzed by western blot. From Figure 8D, we found that cisplatin inhibited phosphorylation of ERK in Siha cells, however, suppression of TROP2 expression by siRNA-1100 induced even lower pERK1/2 expression, while the total ERK1/2 was unaffected. Then we used specific ERK inhibitor U0126 to determine the influence of ERK1/2 activity on cell viability. 10 µM U0126 was used for 2 h prior to cisplatin treatment, the results showed that U0126 effectively strengthened TROP2- siRNA mediated cisplatin sensitivity of Siha cells (Figure 8E). These data indicated that down -regulation of TROP2 expression could significantly enhance chemosensitivity of cervical cancer cells to cisplatin.

## Discussion

As a calcium signal transducer, TROP2 has become a recent focus in tumor research. There are increasing evidences show that TROP2 overexpression is significantly associated with an aggressive malignant phenotype and poor prognosis in various cancer tissues [12]. It is worth noting that Okabe et al. found there was TROP2 overexpression in hepatic oval cells, which can differentiate into hepatocytes and cholangiocytes in injured liver and considered to be facultative hepatic stem cells which decidedly associated with tumorigenesis [13]. However, the expression of TROP2 in cervical cancer and its potential clinical significances have not been described.

In present study, we characterized the expression pattern of TROP2 via immunohistochemistry in 106 cervical cancers, 34 CIN and 20 normal cervical tissues. The results showed that TROP2 was highly expressed in cervical cancer tissues (88.7%), but relatively low expression was found in normal (45%) and CIN tissues (64.7%) (P<0.001), and the percentage of TROP2 - positive cells increased progressively from CINI to CINIII, suggesting that TROP2 protein expression was significantly associated with the cervical oncogenesis and the development of cervical cancer. Moreover, the data of present study showed that the expression of TROP2 was tightly related with lymphatic metastasis and histological grade,patients with TROP2-positive staining exhibited a significantly decreased overall survival and progression free survival, it was also considered to be an independent prognostic predictor for cervical cancer patients according to multivariate analysis. The presented data herein is consistent with previous reports suggest that TROP2 may play a substantial role in tumor biological aggressiveness and metastasis [14], and could be a promising immunotherapy target for further study. Nevertheless, the role of TROP2 in cancer pathogenesis is still considered enigmatic. Cancer cells proliferation is one of the main reasons for the poor prognosis of cancer patients. E Guerra et al. discovered that TROP2 was widely expressed in a variety of human cancers compared with their tissues of origin, and the overexpression was also considered to be necessary and sufficient to quantitatively stimulate tumor growth [15]. In order to determine the relation between TROP2 expression and cell proliferation, we also examined the expression of Ki-67 in the same specimens of cervix tissues. Ki-67 is a proliferation-related nuclear protein to characterize malignant lesions of numerous cancers including cervical cancer [16]. Immunohistochemical assay showed a significant correlation between TROP2 and Ki67 expression, which means TROP2 over expressed in highly proliferative cells and its expression closely correlates with the aggressive behavior.

To better understand the potential connection between TROP2 expression and the biological features of cervical cancer cells, we studied the effect of gain-or-loss of TROP2 expression through ectopic over-expression or RNAi mediated knockdown. In accord with the results obtained in immunohistochemical analysis, vitro study also showed that TROP2 was able to promote cell proliferation by inhibiting cell apoptosis and accelerating cell cycle progression. Evasion of apoptosis is considered to be one of the major hallmarks of tumor development. Bcl-2 and Bax are two critical regulators of cell apoptosis and play pivotal roles in anti-apoptotic and pro-apoptotic, respectively [17]. Our study showed that the overexpression of TROP2 inhibited apoptosis and increased bcl-2 expression, together with decreased bax expression in human cervical cancer cells. The results suggested that TROP2 may facilitate cervical cancer cells to escape the surveillance system by inhibiting cell apoptosis, the effect may be conducted via bcl-2 family activation.

Using PI staining, down-regulation of TROP2 in Siha and CaSki cells induced the cells to accumulate in G1 phase but reducing the cells in the S phase, while enhanced TROP2 expression was required for the growth of HeLa and C33A cells through promoting G1-S transition. A tentative reason might be attributed to certain TROP2 -dependent signaling events that promote up-regulation of growth factors or the of down-regulation growth inhibitory factors, so we further analyzed several cell cycle regulators expression in the four cervical cancer cell lines. CyclinE/CDK2 complex are regulators at the late G1 stage and play an important role in the initiation of DNA replication [18], while cyclinD1 could promote progression from G1 to S phase by activating CDK4 and its amplification has been demonstrated in several human tumors [19]. As a cyclin-dependent kinases inhibitor, P27 negatively regulates G1–G2 cell cycle progression by binding to and preventing the activation of cyclinD1-CDK4 or cyclinE-CDK2 complexes [20]. We found that the changes of cell cycle distribution mediated by TROP2 knockdown is accompanied by reduced expression of cyclin D1, cyclin E, CDK4, CDK2 and increased expression p27, while overexpression of TROP2 exhibited the opposite effect on cell cycle regulators expression. Based on these data, we speculated that the tumorigenic function of TROP2 was due to promoting cell cycle progression and proliferation.

Our immunohistochemical assay showed that TROP2 expression was significantly related with lymphatic metastasis, suggested that TROP2 may be involved in invasion and metastasis of cervical cancer. Tumor metastasis is a complex and multistep process including cancer cell migration, adhesion and invasion and so on. By wound healing and transwell invasion assay, we found that TROP2 has some putative activities in promoting the invasive capability of tumor cells. E-cadherin is a calcium- dependent cell adhesion molecule, plays a considerable role in the formation of adhesion connections and the maintenance of epithelial cell function [21]. In a number of tumors, the loss of cell to cell adhesion mediated by E- cadherin may occur concomitantly with the tumor metastasis and invasion [22]. In present study, we found that up-regulation of TROP2 was sufficient and necessary for decreasing the expression of E-cadherin. Therefore, we speculated that the high levels of TROP2 in cervical cancer cells might induce cell migration and invasion by limiting the expression of E-cadherin.

Many researchers speculated that TROP2 might be a cell-surface signal transducer based on the finding that the cytoplasmic tail domain of TROP2 contains several potential phosphoinositide-binding sites. Recently, the regulatory network of TROP2 was seriously studied by S Alberti et al [23], they found that TROP2 expression was closely related with several important transcription factors such as TP63/TP53L, ERG, GLIS2, FOXM1 and so on, and TROP2 upregulation was shown to stimulate the cancer related downstream targets including Jun, NF- kB, Rb, STAT1 and STAT3. Rafael Cubas et al. speculated that the transient rise in intracellular calcium [Ca2+] mediated by TROP2 may play an important role in the activation of signaling pathway [7]. Therefore, we sought to determine the possible signaling mechanisms involved in TROP2 overexpression triggered proliferation and invasion. It is well documented that activation of MAPK signaling pathway promotes a myriad of cellular activities such as proliferation, migration, invasion and cell survival in multiple various malignancies [24]. ERK1/2 is the canonical member of the MAPK signaling pathway, the phosphorylation and activation of ERK1/2 is involved in mediating tumor progression and metastasis. In present study, western blot analysis proved that the expression of pERK1/2 was notably elevated upon TROP2 up-regulation, but the level of total ERK1/2 was not changed. Elevated expression of pERK1/2 accompanied by increasing its downstream targets cyclin D1 and cyclin E expression, could promote G1-S and G2-M transitions. Given these evidences, we speculated that TROP2 down-regulation may represent a promising approach for cervical cancer treatment by inhibition of ERK1/2-MAPK signaling pathway and its downstream genes.

Furthermore, we determined the association between TROP2 expression and CDDP sensitivity in cervical cancer cells. The results showed that down-regulation of TROP2 sensitizes Siha and CaSki cells to cisplatin treatment by decreased activity of pERK1/2. Using specific ERK inhibitor U0126 effectively strengthened TROP2- siRNA mediated cisplatin sensitivity. Theses finding indicated the role of ERK1/2 pathway in TROP2-siRNA mediated cisplatin sensitivity.

Major progress in immunotherapy targeted TROP2 has been achieved in lots of cancers.

Recent studies demonstrated that a humanized version of TROP2-specific monoclonal antibody (hRS7) labeled with a radioiodinated, diethylenetri-aminepentaacetic acid-appended peptide, ^131^I-IMP-R4, exhibited a desirable therapeutic effect in a breast cancer model in vivo [25]. A new study found that several biologically aggressive cell lines with high expression of TROP2 were susceptible to hRS7-mediated antibody-dependent cell-mediated cytotoxicity (ADCC) by NK cells [26]. In addition, hRS7 conjugated with drugs exhibited more specific antitumor effects against human gastric adenocarcinoma cells [27].

In conclusion, we have demonstrated that the elevated expression of TROP2 in cervical cancer contributes to aggressive phenotypes and poor survival. Meanwhile, silencing TROP2 expression in cervical cancer cells suppresses the proliferation and invasion, also sensitizes cells to cisplatin and induces cell cycle arrest by suppression of ERK1/2 signaling pathway. Therefore, TROP2 has potential as an attractive prognostic marker and a new target for cervical cancer treatment.
